# The impacts of forest management strategies for woodland caribou vary across biogeographic gradients

**DOI:** 10.1371/journal.pone.0170759

**Published:** 2017-02-24

**Authors:** Victoria M. Donovan, Glen S. Brown, Frank F. Mallory

**Affiliations:** 1 Department of Biology, Laurentian University, Sudbury, Ontario, Canada; 2 Wildlife Research and Monitoring Section, Ministry of Natural Resources & Forestry c/o Trent University, DNA Building, 2140 East Bank Drive, Peterborough, Ontario, Canada; Centre for Cellular and Molecular Biology, INDIA

## Abstract

Loss or alteration of forest ecosystems due to anthropogenic activities has prompted the need for mitigation measures aimed at protecting habitat for forest-dependent wildlife. Understanding how wildlife respond to such management efforts is essential for achieving conservation targets. Boreal caribou are a species of conservation concern due to the impacts of human induced habitat alteration; however the effects of habitat management activities are poorly understood. We assessed the relationship between large scale patterns in forest harvesting and caribou spatial behaviours over a 20-year period, spanning a change in forest management intended to protect caribou habitat. Caribou range size, fidelity, and proximity to forest harvests were assessed in relation to change in harvest patterns through time and across two landscapes that varied widely in natural disturbance and community dynamics. We observed up to 89% declines in total area harvested within our study areas, with declining harvest size and aggregation. These landscape outcomes were coincident with caribou exhibiting greater fidelity and spacing farther away from disturbances at smaller scales, hypothesized to be beneficial for acquiring food and avoiding predators. Contrary to our expectation that the large scale maintenance of habitat patches would permit caribou to space away from disturbance, their proximity to harvest blocks at the population range scale did not decrease through time, suggesting that movement toward landscape recovery for caribou in previously harvested regions will likely stretch over multiple decades. Caribou spatial behaviours varied across the two landscapes independently of forest management. Our study underlines the importance of understanding both changes in industry demands, as well as natural landscape variation in habitat when managing wildlife.

## Introduction

Anthropogenic disturbances in forested regions have altered habitat conditions for many wildlife species. Impacts to wildlife may include altered behavioural patterns [1,2], decreased abundance [3], and extirpation from disturbed regions [4], leading to an overall loss in biodiversity [5,6]. In managed forests, the fragmentation of habitat is frequently identified as having negative impacts to wildlife [7,8]. Forest management strategies may include adjusting the spatial organisation of forest harvests in an effort to maintain habitat connectivity and patch size; however, the response of wildlife and effectiveness of these strategies in unclear.

Boreal caribou (*Rangifer tarandus caribou*) is an iconic species of Canada’s boreal forest that has received a great deal of attention due to its vulnerability to extirpation following forest landscape alterations that lead to shifts in forage availability and predator-prey balance [9–11]. Strong connections have been made between boreal caribou’s range loss and the expansion of forest harvesting [4,12]. Evidence suggests that forest harvests create habitat for the alternate prey of caribou’s predators, increasing predator abundance while simultaneously fragmenting caribou habitat, making caribou more susceptible to predation (e.g. *Canis lupus*, genus *Ursus;* [10, 13–16]). Harvests can reduce the availability of large tracks of continuous habitat that permit caribou to space away from predators [17]. Lowering the level of fragmentation associated with forest harvesting is expected to reduce the impacts of harvest disturbance to caribou [11].

Previous studies have documented caribou behavioural response to forest harvests over short time scales ranging from 2 to 6 years (e.g.[18–21]) or in relation to the introduction of forest harvesting on a previously undisturbed landscape (e.g.[22]); however, there is limited understanding of how caribou respond to changing harvesting patterns over multiple decades and across landscapes that vary in natural disturbance regimes. We assessed caribou response to habitat management in Ontario, Canada using telemetry locational data sets and forest harvest records spanning 20 years. We quantified changes in forest harvest area and configuration, and the relationship of harvest to home range size, summer range fidelity, and the proximity of caribou to these disturbances. We tested whether there were differences in the response of caribou to management between two landscapes that differed in fire cycle [23], forest community structure, and climate. The purpose of our study is to assess how caribou spatial behaviours change in response to different harvesting patterns over a large temporal scale and how these changes differ between landscapes.

## Materials and methods

### Study areas

Harvesting patterns and caribou behaviours were assessed in two landscapes within boreal caribou range in Ontario: the northeastern James Bay Lowlands Region and the Northwestern Boreal Shield Region. The northeastern study area is flat with a mean elevation of 250 m [24,25]. The altered humid continental climate, which displays maritime climate characteristics, as well as poorly drained soils, lead to high levels of plaudification [26,27]. Peatlands and monospecific black spruce (*Picea mariana*) stands are dominant habitats throughout the region [25,26,28]. Fire cycles (the time needed to burn an area equivalent to the region of interest) are long, estimated at 398 years, while mean stand age is approximately 148 years [23]. In contrast, the northwestern study area is dominated by well drained soils and rolling hills [29–31]. The climate regime in this region matches the majority of Ontario as humid continental [27]. Jack pine (*Pinus banksiana*) is the dominant stand type, with a mean stand age of 99 years and a relatively short fire cycle of approximately 74 years [29,32,33].

Study area boundaries were defined using caribou locational data collected by the Ontario Ministry of Natural Resources and Forestry (OMNRF) between 1995 and 2013 in relation to the northern limit of forest harvesting.

### Forest harvest assessment

Harvest blocks made each year between 1991 and 2011 were obtained from the OMNRF as polygon shape files and mapped in ArcGIS version10 (www.esri.com). Forest management in Ontario uses 5 year operating plans, where the cutting of one designated harvest area is completed over multiple years [34,35]. Annual harvest polygons were aggregated into a 5-year grouping on a sliding scale (e.g. the 1991 to 1995 aggregated grouping would represent 1995 harvest in our analysis), to provide a more accurate representation of harvesting outcomes based on 5-year management plans. Patterns in forest harvests were quantified using harvest mean patch size and the Clumpiness Index in FRAGSTATS 4.2 [36]. Clumpines Index represents patch clustering, with values ranging from -1 to 1, where a value of -1 indicates maximum disaggregation and 1 indicates maximum aggregation. Total area harvested was also calculated. Changes in all metric values were graphically assessed for temporal trends.

### Telemetry data processing

We compiled 3 different telemetry datasets collected by the OMNRF for adult female boreal caribou. There were an inadequate number of males available to include in our analysis; however, because females are a strong determinant of population fecundity, we were primarily interested in female response to habitat management. Separate ARGOS data sets were collected by the OMNRF for caribou in our western (years from 1995 to 2000, n = 34) and eastern (years from 1998 to 2001, n = 30) study areas. GPS data from adult female caribou between 2009 to 2013 (n = 120) was obtained from collars deployed by OMNRF in support of the Ontario Caribou Conservation Plan [35]. Details of capture and animal handling procedures conducted by OMNRF are described elsewhere [28,33,37], and involved herding caribou into ground nets or use of net gunning from a helicopter. GPS data sets collected from 2009–2013 were more spatially extensive than the older ARGOS data (1995–2001), so we removed individuals from this data set which did not overlap with ARGOS data sets. Similarly, caribou whose cumulative ranges did not overlap with managed forests were removed from our analysis. Following editing, telemetry data sets included locational points from 91 adult female boreal caribou. Early management period data (defined below) were composed solely of Service Argos telemetry locations, while late management period data (defined below) were composed of GPS locations. ARGOS data use the quality of satellite reception to grade each calculated location using Location Classes, with 3 being the highest, followed by 2, 1, 0, A, B, and Z. All ARGOS data were preprocessed by removing data with a Location Class less than 1, as well as any aberrant data found to be at unrealistic distances from other locations. We estimate that most GPS data were within +/- 30 m based on calculations of horizontal error obtained from datalog files on physically retrieved collars.

We defined each biological year as being from May 1 to April 31 the following year, consistent with the approximate start of the calving season. Seasonal periods included: Winter (November 16^th^ to February 15^th^), Spring (February 16^th^ to April 30^th^), Summer (May 1^st^ to September 15^th^), and Fall (September 16^th^ to November 15^th^). These designations were based on current caribou literature within and surrounding each study area [33,38,39].

### Caribou behavioural assessments

**Fig 1:**
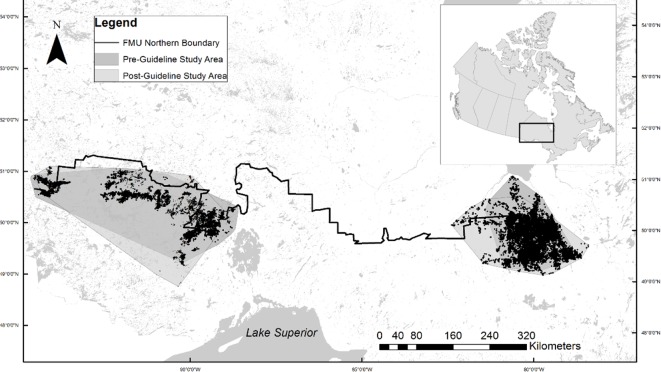
Study Areas. The study areas in eastern and western Ontario, Canada based on boreal caribou (*Rangifer tarandus caribou*) radio-locations (black dots) that occurred in early (prior to wide spread caribou habitat management policy application, 1995–2001) and late (following habitat management policy application, 2009–2013) time periods. Only caribou who had locations that occurred below the northern limit of forest management units (FMU) were assessed. Black tick marks represent 1° parallels on the y axis and 5° meridians on the x axis.

For behavioural assessments, caribou locations were divided into four sub-categories based on time period (early management period between 1995–2001 and late management period between 2009–2013) and study area (eastern Ontario and western Ontario; Fig 1). Time periods represent two different harvesting strategies for caribou. In the early period, habitat management in caribou range (both study areas) primarily focused on moose (*Alces alces*), as provincial caribou habitat management policy was not in place [40]. Such management strategies created small, disconnected blocks of mature forest interspersed with young forest, thought to be detrimental to caribou [34,40,41]. During the late period, a mosaic management approach was used which aimed to maintain large tracts of continuous caribou habitat by aggregating forest harvests on the landscape to reduce harvesting induced habitat fragmentation [42]. Each spatial behaviour was calculated for each region-period class.

We used the 90% contour of fixed kernel utilisation distributions to calculate annual home range size, using the reference smoothing factor (h_ref_) in the ‘AdehabitatHR’ package in R software [43]. Kernel home range estimators are commonly used (e.g.[44,45]) to generate utilisation distributions that provide a more accurate representation of wildlife space use by incorporating an animal’s probability of occurrence at each point in space [46]. Only animals with a minimum of 50 locational points per year were used to estimate annual caribou home ranges, as this number has been shown to be the point at which range size estimates stabilize [46,47]. To handle a large level of over smoothing in late period range estimates, we applied a bootstrapping method, where we calculated the home range for 65 randomly selected sub-sampled GPS locations and then averaged range size over 1000 iterations for each individual within each year. Sub-sampling within large GPS data sets has been shown to have high comparability with lower quality data sets of smaller sample sizes [48].

Caribou have been shown to avoid harvest blocks [19,22], with evidence that harvests less than 10 years of age may be associated with caribou extirpation [12]. We created a proximity index to determine caribou proximity to harvests by taking the ratio of observed to expected distances from harvests made within approximately 10 years of each recorded caribou location during the summer season. The ‘near tool’ in ArcGIS was used to measure the distance of the closest forest harvest block to each caribou location. Expected distances were measured using the systematic approach outlined by Benson [49], where the mean distance to harvest was calculated using 25 m resolution distance rasters created in ArcGIS at both population and individual annual home range scales. Population scales were expected to be representative of caribou’s ability to space away from harvests within our study population’s range, while individual annual home range scales were expected to be representative of an individual’s ability to space away from harvests within its home range. The population range was created by using the 100% Minimum Convex Polygon (MCP) of all caribou locations in each data group (e.g. east early, west early etc.) and adding a 7.5 km buffer to each range. Annual ranges were created for each individual using the 90% kernel utilization distribution, with expected annual distances calculated for each individual within each year.

Caribou display strong fidelity to summering ranges [50–52]. To assess the influence of harvesting on such behaviour, we created a summer fidelity index. The fidelity index was calculated as the ratio of the average distance between paired animal locations and the average distances expected under a null hypothesis of no fidelity. Animal locations recorded on the same day during consecutive years (i.e., July 1^st^ 1998 and July 1^st^ 1999) were paired and the distance was measured between each pair of points [49, 51]. Distance calculations between paired points were calculated following Popp et al. [52]. Paired distances were then averaged for all pairs of locational points measured for each caribou [50,52]. Pairing animal locations by day, as opposed to pairing all possible combinations of locations within a weekly or monthly period, was deemed more appropriate for our dataset and facilitates comparisons with previous studies that have used the technique. The ARGOS and GPS collars used to collect our data varied among the original studies in the location collection schedule with respect to the calendar date and time intervals between locations; however, within each dataset there was consistency among years in the calendar days on which locations were collected. Only individuals with a minimum of 10 locational points spread across each month of the summer season were included in the analysis. Null or expected distances for each region-period class were derived using the average distance between all possible pairs of locations for all collared caribou within each annual summer season [50]. These values were averaged over all measured years within each region-period class (east early, west early, etc.). We then created a ratio of expected to paired distance values to represent fidelity index in subsequent analysis. By using this ratio, we created a relative measure which incorporated a null distance expectation specific to each region-period class (east early, west early etc.).

Both individual annual home range scale proximity index and summer fidelity index assessments were meant to represent small scale caribou response to forest harvest within their selected range. Population level proximity index and annual home range size were meant to represent caribou response to harvesting at larger, landscape scales.

Because individual caribou were exposed to varying levels of harvesting disturbance, we used an individual-based analysis approach and measured a range of harvest covariates surrounding each caribou’s telemetry locations. This also allowed us to isolate the influence of varying levels of disturbance on caribou behaviour (summarized in Table 1).

**Table 1:** Caribou behaviour model covariates.

Variable Abbr.	Definition	Behavioural Model
Group	A categorical variable representing the region-period classes: Early East, Early West, Late East, Late West.	• Home Range • Fidelity • Proximity
CutinHR	The percent area composed of forest harvests under 15 years of age from the recorded caribou locations in an individual’s 90% fixed kernel home range.	• Home Range • Proximity
CutHRBuffer	The percent area composed of forest harvests made within 15 years of the recorded caribou locations within a 21 km (west) or 37 km (east) buffer region surrounding an individual’s 90% fixed kernel home range. Buffer distances were calculated using the square root of the average annual 100% Minimum Convex Polygon (MCP) home range for caribou in the eastern and western Ontario regions.	• Home Range • Proximity
CutBuffer	The percent area composed of forest harvest within the 7.5 km buffer region surrounding the forest harvest nearest to a measured caribou location. Buffer distance was based on Lesmerises et al., [53], who found that caribou will make decisions about a habitat patch based on the surrounding landscape matrix 7.5 km away.	• Proximity
CutPoint	The average area composed of forest harvest within a 12 km (west) or 21 km (east) buffer region surrounding each locational point included in fidelity measures. Because buffers were circular, buffer distances were calculated by dividing the average 100% MCP home range size for caribou in the eastern and western study regions by π and taking the square root of this value.	• Fidelity

An overview of model covariates used in candidate models run in Akaike’s Information Criterion for small sample sizes to explain variation in annual home range size, population and annual scale proximity index and summer fidelity.

### Statistical assessments

Linear mixed effects models from the ‘nlme’ package in R were used to assess temporal changes in proximity and home range size between early (prior to wide spread caribou habitat management policy application, 1995–2001) and late (following habitat management policy application, 2009–2013) time periods [54]. Each spatial behavioural metric was used as a dependant variable, region-period class (west early, east early, etc.) and harvest measures were included as fixed effects and individual was used as the grouping factor for random effects. In order to meet model assumptions, we applied relevant correlation and variance structures within our models where necessary. For fidelity, we modeled changes using linear regression, as fidelity measures were averaged over all years for each individual, with the same fixed effects described for linear mixed effect models. For all models, square root transformations were applied where needed to fit normality assumptions.

In all analyses, AICc (Akaike’s Information Criterion for small sample sizes) from the package “MuMIn” in R was used to select between candidate models including different combinations of harvest measures (Table in S1 Table; [55]). Models with ∆AIC<2 were model averaged using “MuMIn”. Once the top model was selected, post-hoc tests were conducted where necessary using the ‘glht’ function from the package “multcomp” in R [56].

## Results

### Harvest assessment

**Fig 2:**
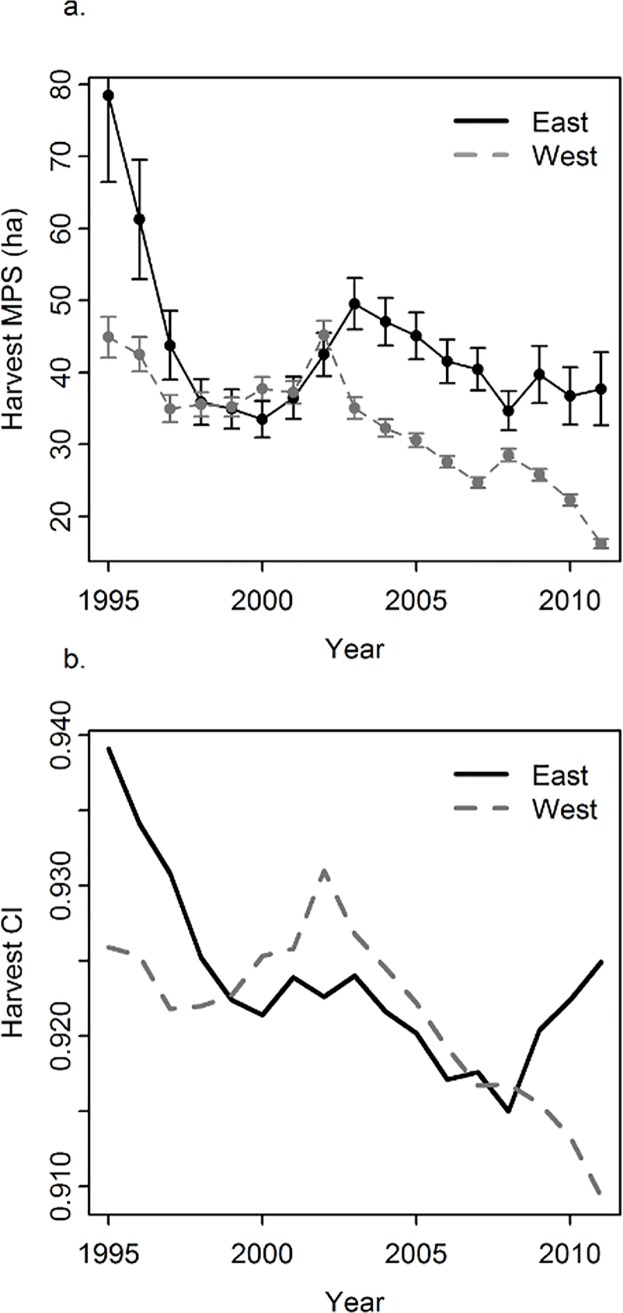
Harvest assessment trends. The change in harvest Mean Patch Size (Harvest MPS) and harvest Clumpiness Index (Harvest CI) through time in both the western (grey, dashed line) and eastern (black, solid line) study areas. Caribou habitat management introduction occurred in 1999 (black, dotted line). Error bars represent standard error.

Harvest mean patch size and Clumpiness Index declined through time in both eastern and western study areas (Fig 2). Mean harvest patch size was greater in the eastern study area compared to the west. Total area harvested within each study region declined dramatically through time in both study areas: 77% in the western study area and 89% in the eastern study area from 2002 (the peak total harvest date for both study areas) to 2011 (the last measured year).

### Behavioural assessments

**Fig 3:**
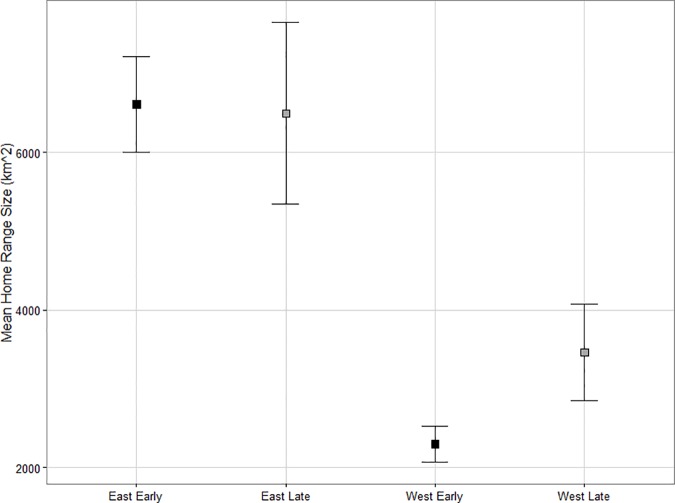
Mean caribou home range sizes between time periods and study areas. A comparison of mean home range size for boreal caribou (*Rangifer tarandus caribou*) between the eastern and western study areas in the early period (black; prior to wide spread caribou habitat management policy application, 1995–2001) and late period (grey; following habitat management policy application, 2009–2013). Error bars represent standard error.

The percentage of harvest area within annual home ranges and in the buffered zones around home ranges were the most important variables in influencing home range size (Table 2A). Home range size decreased with increasing percentage of harvest within home ranges (p = < 0.01, β = 46.67, 95% CI [26.66, 66.68]), and with increasing percent harvest area surrounding a caribou’s home range (p = <0.01, β = -22.64, 95% CI [-38.32, -6.96]). There was no significant change in home range size in either study area between time periods (east: p = 0.86, β = 10.38, 95% CI [-23.71, 44.47], west: p = 0.64, β = -16.49, 95% CI [-52.21, 19.22]); however, eastern Ontario was found to have significantly larger home ranges than western Ontario in both the early and late time periods (early: p = <0.01, β = -62.27, 95% CI [-98.01, -26.53], late: p = <0.01, β = -89.15, 95% CI [-122.36, -55.94]; Fig 3), with an average home range size of 7430.78 ± 573.71 km^2^ compared to 3042.84 ± 413.27 km^2^, respectively.

**Table 2:** Akaike’s Information Criterion for small sample size (AICc) outputs for the top 5 candidate models for behavioural metrics.

Model	df	LogLik	AICc	∆AICc	W
*a. Home Range Size*					
Group+ CutinHR+ CutHRBuffer	9	-999	2017	0	0.89
Group+ CutinHR	8	-1002	2021	4	0.09
Group+ CutHRBuffer	8	-1004	2025	8	0.01
Group	7	-1008	2031	13	0
Null	4	-1035	2079	63	0
*b. Proximity Index*, *Population*					
Group+CutinHR	9	29035	-58053	0	1.00
Group+CutinHR+CutHRBuffer	10	29001	-57983	69	0
Null	5	28983	-57957	95	0
Group	8	28956	-57897	155	0
Group+ CutinHR+CutBuffer	10	28938	-57857	195	0
*c. Proximity Index*, *Annual*					
Group+ CutBuffer	9	24858	-49698	0	0.87
Group+ CutBuffer+CutinHR	10	24857	-49694	3	0.13
Null	5	24799	-49588	109	0
Group	8	24800	-49584	114	0
Group+CutinHR	9	24798	-49579	118	0
*d. Summer Fidelity*					
Group	5	3	4	0	0.62
Group+CutPoint	6	4	5	1	0.38
Null	2	-27	59	54	0

The top 5 candidate models (variable explanations in Table 1) explaining variation in home range (HR) size (a), population (b) and annual (c) proximity index, and summer fidelity (d) for caribou exposed to changing management in Ontario: the degrees of freedom of each model (df), the natural logarithm of maximum likelihood for each model (LogLik), the Akaike's information criterion adjusted for small sample size bias (AICc), the change in AICc (∆AICc), the Akaike weight for each model (W), and variable importance values (i) included when model averaging was applied.

The amount of harvest within a caribou home range influenced caribou proximity to harvest at the population scale (Table 2B). Caribou proximity index increased with increasing percent harvest area within their home range (Population: p = 0.03, β = 0.05, 95% CI [0.01, 0.10]), suggesting that animals spaced themselves farther from harvests when there was a higher level of harvesting within their home range. At the annual scale, proximity to harvest was related to the level of harvesting that surrounded the nearest harvest patch (Table 2C). Caribou were closer to a harvest than expected when that harvest had a high level of harvesting surrounding it (p = <0.01, β = -0.05, 95% CI [-0.06; -0.04]).

**Fig 4:**
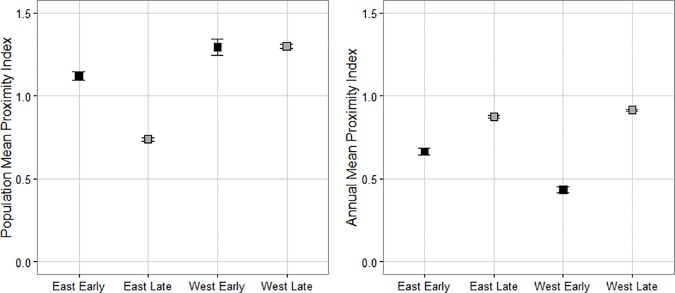
Mean caribou proximity index values between time periods and study areas. A comparison of mean proximity index values for boreal caribou (*Rangifer tarandus caribou*) between the eastern and western study areas in the early period (black; prior to wide spread caribou habitat management policy application, 1995–2001) and late period (grey; following habitat management policy application, 2009–2013) at the population and individual annual home range scales. Error bars represent standard error.

There was no change in caribou proximity index between early and late time periods in eastern or western Ontario at the population scale (p = 0.40, β = -0.17, 95% CI [-0.55, 0.22]; p = 0.98, β = -0.07, 95% CI [-0.60, 0.45] respectively). In the west, mean proximity index ratio of 1.30 ± 0.01 suggests caribou were farther from harvests than expected, while in the east, caribou were closer to harvests than expected, with a mean proximity index ratio of 0.79 ± 0.01. At the annual scale, there was no change in caribou proximity index between time periods in eastern Ontario (p = 0.30, β = 0.10, 95% CI [-0.05; 0.26]), with a mean proximity index value of 0.85 ± 0.01 suggesting caribou were closer to harvests than expected. However, caribou became farther from harvests during the late period of study in western Ontario (p = <0.01, β = 0.23, 95% CI [0.08; 0.38]; Fig 4), with mean proximity index values moving from 0.43 ± 0.01 to 0.91 ± 0.02.

**Fig 5:**
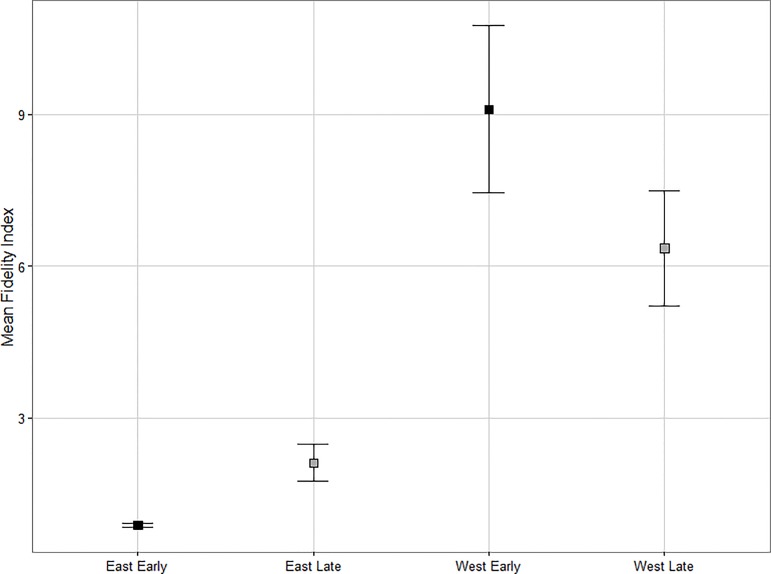
Mean caribou fidelity index values between time periods and study areas. A comparison of mean fidelity index values for boreal caribou (*Rangifer tarandus caribou*) between the eastern and western study areas in the early period (black; prior to wide spread caribou habitat management policy application, 1995–2001) and late period (grey; following habitat management policy application, 2009–2013). Error bars represent standard error.

The harvest and region-period class variables had importance values of 0.38 and 1.00 respectively, in explaining variation in summer fidelity behaviour (Table 2). There was no change in caribou fidelity to summering areas in the western study area between time periods (p = 0.11, β = -0.17, 95% CI [-0.38, 0.04]). In the eastern study area, we found a significant increase in fidelity to summering areas (p = <0.01, β = 0.32, 95% CI [0.14, 0.51]; Fig 5) with mean fidelity index values of 0.87 ± 0.05 in the early period compared to 2.11 ± 0.36 in the late period. Fidelity index values below 1 in the early period indicate that caribou were farther from previously used locations than expected. This suggests that no fidelity behaviour was displayed during the early period in the east. Western caribou displayed much stronger fidelity to summer ranges than eastern caribou in both early and late time periods (p = <0.01, β = 0.94, 95% CI [0.74, 1.15]; p = <0.01, β = 0.46, 95% CI [0.26, 0.65] respectively; Fig 5) with an average western fidelity index value of 7.53 ± 0.98.

## Discussion

Caribou behaviours varied significantly between the two study landscapes, suggesting a broad scale adaptive response to habitat heterogeneity. These results are not atypical; previous research has documented an array of wildlife populations which display different spatial behavioural responses among landscapes (e.g. [11,57,58]). Western Ontario caribou had much smaller home range areas than eastern Ontario caribou, and also displayed much stronger summer fidelity behaviour. Our modeling effort associated smaller home range size with higher levels of harvesting within and surrounding a caribou’s home range. Animals tend to have smaller home ranges where higher quality habitat is available, meaning they do not need to travel as widely to fulfil their needs [59]. Western Ontario may provide better quality habitat, regardless of the elevated harvesting levels we observed in comparison to the eastern study area. However, such patterns could also be indicative of higher levels of historical fragmentation in the western population range. Fragmentation can compromise caribou movement [60] and western Ontario caribou range has historically had much greater overlap with harvesting activities [40] as well as been more frequently disturbed by wildfire [32]. Differences in disturbance regimes and habitat distributions likely similarly shape differences in predator and alternative prey distributions between landscapes [14,15]. The differences we observed in caribou behaviour are thus likely related to a number of interacting factors related to habitat distribution and abundance within each landscape as well as differences in natural and anthropogenic disturbance regimes (e.g. harvesting, mining) between regions [23,27,32,40].

Forest harvests were shown to have a strong impact on caribou spatial behaviour. The influence of harvesting on home range observed in both of our study areas suggest that caribou home ranges contract with increasing exterior disturbance, while caribou will increase their home range size with increasing interior disturbance. This matches with previous caribou research which suggests that caribou will initially expand their home ranges to avoid disturbances, however, as the level of fragmentation increases on the landscape, caribou can become ‘trapped’ in smaller patches of habitat, decreasing their home range size [18,21,22,60].

Similar associations between harvests and proximity were observed, with a positive relationship evident between levels of harvesting within home ranges and caribou distance from harvests (increasing proximity index). However, at the annual scale, we observed caribou moving closer to harvests (decreasing proximity index) that had high levels of disturbance in the surrounding landscape. High levels of fragmentation may prevent caribou from spacing away from harvests [60]. Other research suggests that declining home range size and increased association with forest harvests may be a maladaptive response to forest harvesting that compromise a caribou’s ‘spacing out’ strategy [17,22,60] and have been associated with elevated predation risk [14–16,18].

Fidelity, as assessed in our study, did not appear to be strongly influenced by harvesting. It is possible that the extent of habitat disturbance was maintained below levels that might impact this behavioural strategy. Caribou generally demonstrate high summer calving site fidelity, returning to same location every year to calve [20,50,61]. Fidelity behavior is believed to be associated with predation threat; driven by site advantages associated with visibility or knowledge of escape routes from predators [20,50,61]. Thus, investigations into the direct relationship between predator density associated with forest harvests and fidelity behaviour may yield stronger outcomes. Changes in fidelity may also be more related to finer scales of behavioural response or immediate disturbance (e.g. [62]). Similarly, aspects of management other than harvests may more strongly influence fidelity behaviour. For instance, extensive road systems are built to support harvesting activities. Faille et al. [20] found a link between fidelity behaviour and roads, independent of forest harvest patches. Future research could investigate this link in relation to changing management.

Eastern Ontario caribou displayed an increase in fidelity behaviour between our observed time periods. Increasing levels of disturbance have been associated with decreased levels of caribou fidelity [18,20], potentially suggesting that caribou were less disturbed in the later period of study in the east. This matches slight increases in population growth rates estimated for this region (0.88 between 1998 and 2000 to 0.92 in 2012), though recruitment estimates (calves per 100 females), which ranged from approximately 23 to 29 in our early study period, fell between 13 and 20 during our late study period [63]. Under some conditions, increased fidelity might be reflective of a maladaptive behavioural response to harvesting associated with increased fragmentation, where caribou become trapped in smaller patches of habitat close to harvests [60,64]. However, there was no change in the average distance between available summering locations (expected distance) in our eastern study area, suggesting that this mechanism was unlikely. Similar results were seen in annual proximity measures, with increasing caribou distance from forest harvests between time periods in western study region. Caribou that are able to increase their distance from disturbance are likely to reduce harvest associated predation risk [14,18,65]; however, predation rates for this population are unknown.

Small scale caribou behavioural results correspond with the dramatic declines in the total area harvested observed in both study areas. In Ontario, the percentage of available wood approved in management plans that was actually harvested dropped from 73% in 2004 to 41% in 2008 [66]. Along with global economic recession, reduced forest industry productivity may also be associated with the Canada-U.S. Softwood Lumber Agreement in 2006 and a large economic downturn in the United States housing market that occurred in 2007 [66–68]. Based on our spatial behavioural results, we suggest that although we observed decreasing harvest aggregation and smaller harvests, which are suggestive of increased habitat fragmentation by harvests, dramatic declines in total area harvested likely led to overall lower levels of habitat fragmentation and disturbance to caribou at smaller scales.

Our larger scale behavioural measures of population level proximity and annual home range size did not change through time in either study area. This is particularly interesting in the east, where we observed dramatic declines in the amount of harvesting which occurred over the last decade, while proximity values remained below 1 (closer to harvests than expected based on harvest distribution). Caribou in close proximity to harvests are likely exposed to elevated predation risk regardless of changes in harvesting level [14,60,65]. These results could indicate that decreased harvesting was not adequate to alleviate the previous impacts of harvesting fragmentation on caribou space use. However, winter harvesting on lowland black spruce as well as CLAGG (‘Careful Logging around Advanced Growth’) and HARP (‘Harvesting And Regeneration Program’) harvesting methods, used to reduce forest over story while maintaining natural regeneration in understory vegetation, are commonly applied in the east and have been shown to have lower impacts on forest regeneration [69]. Thus, it is possible that caribou remained closer to harvests in the east because harvests posed less of a danger relative to the western study region. Further research into the predation threat associated with different harvesting methods should be pursued.

Our results suggest that large decreases in total forest area harvested likely lowered disturbance to caribou at smaller scales. However, it remains unclear as to whether changes in harvest size and configuration had an impact on caribou spatial behaviour. No changes in large scale behavioural metrics suggest that movement toward landscape recovery for caribou in previously harvested regions will likely stretch over multiple decades. According to recent estimates for portions of our eastern and western study regions, boreal caribou populations in our study regions are still in decline [63,70]. Future research should focus on separating the influence of harvesting levels from harvesting configuration to better understand the individual impacts of each disturbance element. Further, our study underlines the importance of understanding both changes in industry demands, as well as landscape variations in habitat heterogeneity when managing wildlife. These factors need to be considered during both management application and monitoring for effective management.

## Supporting information

S1 Table Candidate models of caribou spatial behaviour. Candidate models for caribou spatial behaviour response to forest harvesting among region-period classes.
(PDF)

S1 File Harvest and caribou behaviour data. A data file containing raw data for caribou behaviour model inputs and annual harvesting data for both study areas.
(XLSX)
