# Supplementary material for: The impacts of forest management strategies for woodland caribou vary across biogeographic gradients
Source: PLoS One. 2017 Feb 24;12(2):e0170759. doi: 10.1371/journal.pone.0170759 (PMC5325202; doi:10.1371/journal.pone.0170759)
Supplement: S1 Table — Candidate models for caribou spatial behaviour response to forest harvesting among region-period classes. (PDF) [file pone.0170759.s001.pdf]

S1 Table. Candidate models of caribou spatial behaviour. Candidate models for caribou spatial behaviour response to forest harvesting among region-period classes.

Home Range Size
Group+ CutinHR+ CutHRBuffer
Group+ CutinHR
Group+ CutHRBuffer
Group
Null
Proximity Index, Population
Group+CutinHR+CutHRBuffer
Group+ CutinHR+CutBuffer
Group+CutinHR
Group+CutBuffer
Group+CutHRBuffer
Group
Null
Proximity Index, Annual
Group+CutinHR+CutBuffer
Group+CutinHR+CutHRBuffer
Group+CutinHR
Group+CutHRBuffer
Group+CutBuffer
Group
Null
Summer Fidelity
Group+CutPoint
Group
Null
